# Subsurface Event Detection and Classification Using Wireless Signal Networks

**DOI:** 10.3390/s121114862

**Published:** 2012-11-05

**Authors:** Suk-Un Yoon, Ehsan Ghazanfari, Liang Cheng, Sibel Pamukcu, Muhannad T. Suleiman

**Affiliations:** 1 Department of Computer Science and Engineering, Lehigh University, Bethlehem, PA 18015, USA; E-Mail: cheng@cse.lehigh.edu; 2 Department of Civil and Environmental Engineering, Lehigh University, Bethlehem, PA 18015, USA; E-Mails: ehg209@lehigh.edu (E.G.); sp01@lehigh.edu (S.P.); mts210@lehigh.edu (M.T.S.)

**Keywords:** subsurface sensing, subsurface event detection and classification, Wireless Signal Networks (WSiNs)

## Abstract

Subsurface environment sensing and monitoring applications such as detection of water intrusion or a landslide, which could significantly change the physical properties of the host soil, can be accomplished using a novel concept, Wireless Signal Networks (WSiNs). The wireless signal networks take advantage of the variations of radio signal strength on the distributed underground sensor nodes of WSiNs to monitor and characterize the sensed area. To characterize subsurface environments for event detection and classification, this paper provides a detailed list and experimental data of soil properties on how radio propagation is affected by soil properties in subsurface communication environments. Experiments demonstrated that calibrated wireless signal strength variations can be used as indicators to sense changes in the subsurface environment. The concept of WSiNs for the subsurface event detection is evaluated with applications such as detection of water intrusion, relative density change, and relative motion using actual underground sensor nodes. To classify geo-events using the measured signal strength as a main indicator of geo-events, we propose a window-based minimum distance classifier based on Bayesian decision theory. The window-based classifier for wireless signal networks has two steps: event detection and event classification. With the event detection, the window-based classifier classifies geo-events on the event occurring regions that are called a *classification window*. The proposed window-based classification method is evaluated with a water leakage experiment in which the data has been measured in laboratory experiments. In these experiments, the proposed detection and classification method based on wireless signal network can detect and classify subsurface events.

## 1. Introduction

Wireless sensor networks have the potential to be implemented in subsurface sensing and monitoring applications, which are called Wireless Underground Sensor Networks (WUSNs) [1]. The key applications could be monitoring subsurface hazards and characterizing subsurface environments in real-time using wireless sensor nodes. In the applications of subsurface hazard monitoring, wireless sensor networks can be used for slope monitoring or predicting landslides [2,3]. Subsurface wireless sensor nodes can adopt existing sensing devices (e.g., MICA2/z with MTS300/310/400/410 for light, temperature, humidity, barometric pressure, seismic, acoustic, sounder, and accelerometer [4]). However, the sensor nodes only provide point measurements and are incapable of providing regional measurement for characterization and monitoring of subsurface medium. To characterize and monitor subsurface environments, the concept of Wireless Signal Networks (WSiNs) is introduced in this paper. WSiNs use the wireless signal strength variation between the distributed sensor nodes as the main indicator of a subsurface event or physical change in the host medium.

The potential subsurface monitoring applications include landslides, earthquakes, and active fault zone monitoring which involve soil movement [1,3]. The monitored events in the applications are characterized by the localization of sensors. This localization allows sensors to estimate their locations using information transmitted by a set of seed sensors. Other potential applications of characterizing subsurface environments include monitoring of oil leakage from subsurface reservoirs, water leakage from underground pipelines, seepage in earth dams, and estimation of soil properties and conditions [5]. The soil properties and conditions such as degree of compaction, gradation, and salinity level can potentially be monitored based on the Received Signal Strength (RSS) information, which are demonstrated in the paper. The received signal strength with respect to distance from the source can be considered as information to be classified for different events. The received signal strength information can be classified by minimum distance classifier using Bayesian decision theory. In the application of Bayesian theory to runtime wireless underground sensor networks, the computational power is an important factor to be considered because wireless sensor nodes have limited computational power. In the paper, a *window-based minimum distance classifier* is proposed for a computationally efficient classifier for wireless signal networks maintaining high accuracy with less computation. The paper aims to make four contributions:

- A novel concept of Wireless Signal Networks (WSiNs) for subsurface event detection and classification based on the underground radio propagation is introduced. The concept is demonstrated through experiments using real wireless sensor nodes. Compared to the existing geo-sensing and monitoring methods, the concept provides both point sensing and regional sensing between the underground sensor nodes in real-time.
- A detailed list of soil properties is provided with experimental data on how radio propagation is affected by soil properties in subsurface communication environments. With the comprehensive analysis of soil properties in subsurface communication environments, network designers and researchers can estimate underground communication radius and network capacity, and use the experimental data for their underground wireless network design.
- The WSiN concept is evaluated for the event detection of subsurface applications such as detection of water intrusion, relative density change, and relative motion using actual underground sensor nodes. For the event detection concept and experiment, a new type of subsurface sensing system is developed.
- A window-based minimum distance classifier based on Bayesian decision theory is evaluated with the water leakage experiment. The received signal strength information over the existing underground communications of wireless sensor nodes is used as a tool for subsurface event detection and classification with high accuracy and less computation.

The rest of the paper is organized as follows: In Section 2, the paper describes existing subsurface monitoring techniques. Section 3 introduces the concept of wireless signal networks and their applications as well as challenges and solutions of wireless signal networks. In Section 4, the paper provides the soil properties affecting received signal strength with experimental data. Then, in Section 5, the paper presents subsurface event detection and classification methods. In Section 6, the paper provides the performance evaluations and discussion. Finally, Section 7 concludes the paper.

## 2. Existing Monitoring Techniques

Electromagnetic (EM) wave propagation has been widely used in soil science as a means of geo-sensing and determination of some soil properties. For instance, moisture content and salinity of soil have been measured using different techniques including four-electrode sensors (surface array or insertion probes), remote electromagnetic induction sensors [6,7] and time domain reflectometric sensors [8]. Also, ground penetrating radar (GPR) and active microwave remote sensing have been implemented in soil moisture detection [9,10].

In general, subsurface monitoring has been accomplished through destructive and non-destructive techniques including direct soil sampling, probing and soundings, and using geophysical mapping tools. Although these techniques have been successfully implemented to characterize the state of geo-media, there are challenges associated with these techniques including difficulties in providing real-time data to track geo-hazard (e.g., landslides, slope failures) and deployment challenges specifically the requirement of wired connections in some conventional techniques.

To address some of these challenges associated with the aforementioned techniques, wireless sensors have been used recently for subsurface monitoring. Current applications of wireless sensors include measurements of earth-slope inclination, landslides, strong ground motion, and soil-structure interactions [11–15]. In the examples cited above the sensors are attached to the exterior of the system of interest and embedded using supplementary frames. These sensors are designed to measure a specific property (point measurement) and transmit the collected data via wireless communication. Recent papers introduced a new approach that uses the characteristics and changes of the wireless signal as an indication of the changes in the properties of the host medium [16–19].

## 3. Wireless Signal Networks

### 3.1. Concept of Wireless Signal Networks

**Figure 1.**
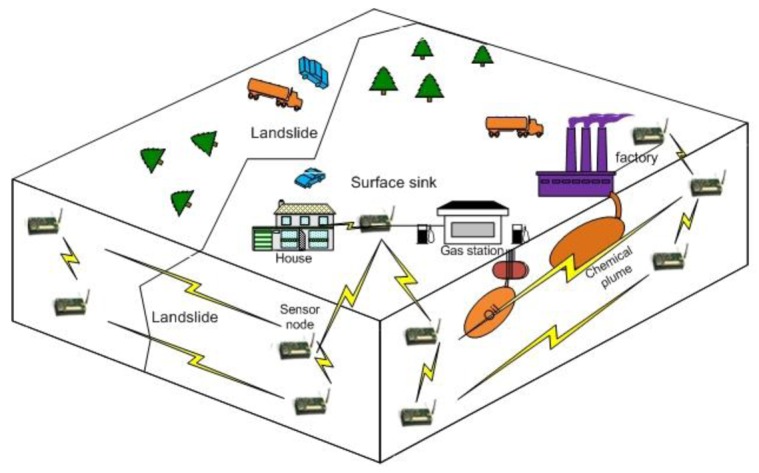
Subsurface monitoring with Wireless Signal Networks (WSiNs).

Wireless Signal Networks (WSiNs) use the radio signal strength variation as the main indicator of an event in the physical domain of the wireless signal network [18]. Soil properties such as density and water/mineral content are known to affect radio wave propagation. When and if these physical properties of the host soil change during the evolution of a geo-event (subsurface event), they in turn affect the transmission quality and strength of the radio waves within the region of the event. Using the new approach, regional subsurface monitoring in real-time can be achieved. Figure 1 shows the concept of subsurface monitoring with wireless signal networks for detection of landslide and chemical plume/oil leakage. The new approach could provide both point sensing and regional sensing between the underground transceivers, and real time measurements that potentially can be used for subsurface monitoring.

### 3.2. Underground Radio Propagation

A free-space radio propagation model can be used to predict the Received Signal Strength (RSS) between the transmitter and the receiver based on the clear and unobstructed line-of-sight (LOS) path between them. A well-known radio transmission formula was introduced by H. T. Friis in 1946 [20]. The received power in free space is given by the Friis free space equation as follows:

(1) $$P_{r}(d)=\frac{P_{t}G_{t}G_{r}\lambda^{2}}{{\left(4\pi \right)}^{2}d^{2}L}$$

where *P_r_* is the received power which is a function of the transmitter-receiver distance, *P_t_* is the transmitted power, *G_t_* is the transmitter antenna gain, *G_r_* is the receiver antenna gain, *d* is the distance between the transmitter and receiver, *L* is the system loss factor not related to propagation (*L* ≥ 1), and *λ* is the wavelength [21].

In geo-media, the signal strength attenuation is much faster than in air due to the transmission power loss in the medium. For lossy dielectrics, the permittivity and electrical conductivity are dependent on the operating frequency. These two properties characterize the displacement (polarization) current and the conduction current which incur the power losses of the electromagnetic wave in the soil [22]. An accurate and simple subsurface wireless signal propagation model for low-power devices (*i.e.*, wireless sensor nodes) at a frequency of 2.4 GHz was developed and its performance was evaluated using real wireless sensor nodes (MICAz) [23]. In the underground radio propagation model, the underground medium has permittivity *ε* = *ε_r_ε*_0_, permeability *μ*_0_, and electrical conductivity *σ*, where *ε*_0_ (8.85 × 10^−12^ F/m) is the permittivity and *μ*_0_ (4π × 10^−7^ H/m) is the permeability of air. In the derivation of the underground radio propagation model, the source is imagined to be a vertical electric dipole of length *ds* and carrying a current *I*. The received signal strength in geo-media (soils) is defined as follows:

(2) $$\begin{array}{c} P_{r}(d)=\frac{A_{\text{eff}}\cos\theta_{\eta }}{2\left|\eta \right|}{\left(\frac{\text{Ids}\mu_{0}\omega }{4\pi }\right)}^{2}\frac{e^{-2\alpha d}}{d^{2}}\\ =K\frac{e^{-2\alpha d}}{d^{2}} \end{array}$$

where *A_eff_* is the effective antenna area of the receiver, $\eta =\sqrt{\frac{i\omega \mu }{\sigma +i\omega ɛ}}$ is the intrinsic wave impedance of the medium, *θ_η_* is the phase angle of the intrinsic impedance *η* = |*η*|*e^jθη^*, *ω* is angular frequency, *d* is the distance from the source, *α* is the attenuation constant of the medium, and $K=\frac{A_{\text{eff}}\;\cos\theta_{\eta }}{2\left|\eta \right|}{\left(\frac{\text{Ids}\omega \mu_{0}}{4\pi }\right)}^{2}$ [23]. Comparing the theoretical estimations of the underground radio propagation and the measured data using MICAz (2.4 GHz), the theoretical model fits the measured data well within a 3.45 dBm deviation or with an accuracy of 96.33% on average [23].

### 3.3. Applications for Wireless Signal Networks

To monitor underground environments, the underground radio propagation model can be used by underground wireless signal networks which use the wireless signal strength variation in the soils as the main indicator of an event. By analyzing the received signal strength, the wireless sensor networks can collect additional information from the wireless data carrier. The targeted events are subsurface hazards such as a landslide or earthquake which involve a lot of shifting and moving of earth masses, and the intrusion of a chemical plume which would dramatically change the physical properties of the host soil as shown in Figure 1. These events would affect the transmission of radio waves and the received signal strength in the region. Because the underground received signal strength deviation is very small, the subsurface events can be detected by the signal strength deviation as follows: *P_r_*(*d*, *t*) ≥ *ζ* × *P_r_*(*d*, *t* − Δ*t*), where *ζ* is the deviation criterion which can be empirically determined based on the underground environments. If the soil condition is changed due to an event such as water leakage on the medium between sensors, the sensor can detect the event based on the decreased received signal strength and classify the event based on the reference data which can be generated by Equation (2) or empirical data.

### 3.4. Challenges and Solutions of Wireless Signal Networks

The deployment of wireless signal networks in underground environments imposes challenges as follows:

#### 3.4.1. Installation and Management

The installation and management of underground sensors are much more difficult than aboveground networks because drilling, sensor embedment and management are not easy tasks. Even if the sensor position is easily traceable; the sensor can be damaged during digging. Thus, before the deployment of underground sensor networks, the network and topology should be designed to minimize installation and management costs. For example, the sensor with high energy consumption can be deployed in shallow depth. To avoid replacing the battery, high capacity batteries and power saving sensor operation can be used. For the sake of minimizing energy consumption of underground sensors operation, efficient sleep mode and long data reporting interval can be adopted.

#### 3.4.2. Communication Radius

**Figure 2.**
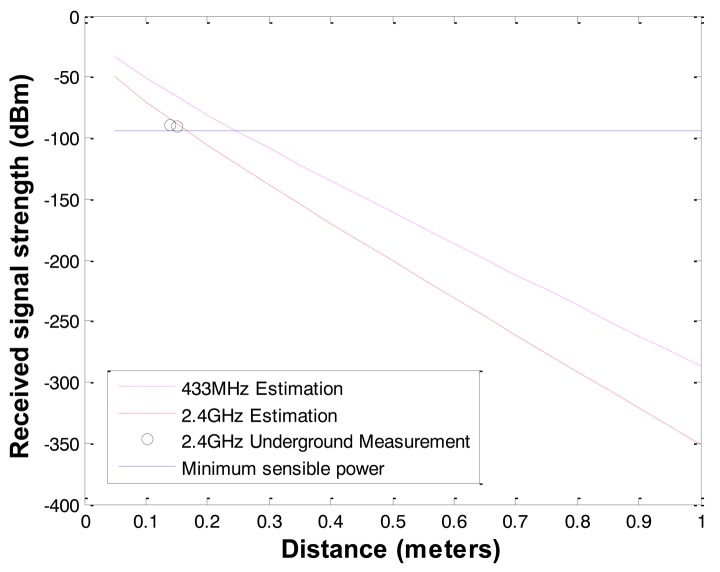
Underground radio signal attenuation with 2.4 GHz and 433 MHz.

The communication radius in soil is much shorter than in the air due the high attenuation of radio propagation in soil medium. Based on the underground radio propagation model and the field measurements; the communication radius of the commercial wireless sensors such as 2.4 GHz MICAz and 433 MHz MICA2 with 1 mW transmission (Tx) power are about 20 cm and 30 cm in wet clay type soil (measured electrical conductivity of 780 mS; estimated relative permittivity of 30) sampled in Lehigh University Goodman Campus as shown in Figure 2.

#### 3.4.3. Low Frequency Wireless Signal Networks

**Figure 3.**
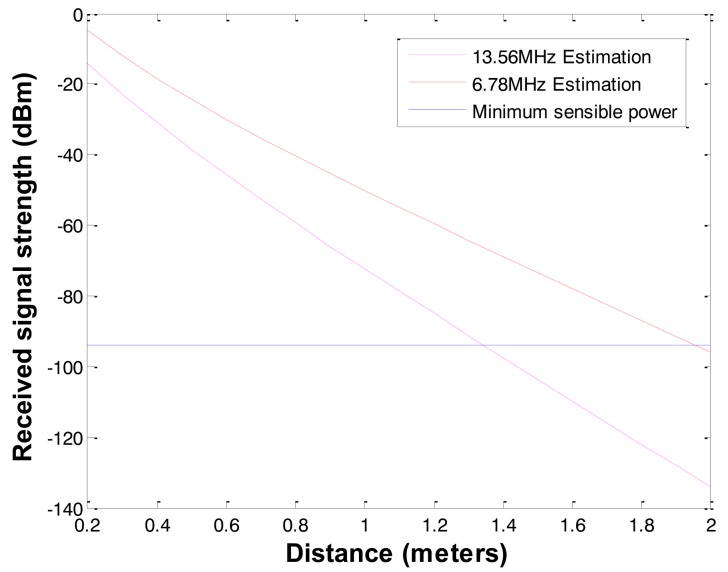
Underground radio signal attenuation with 13.56 MHz and 6.78 MHz.

**Figure 4.**
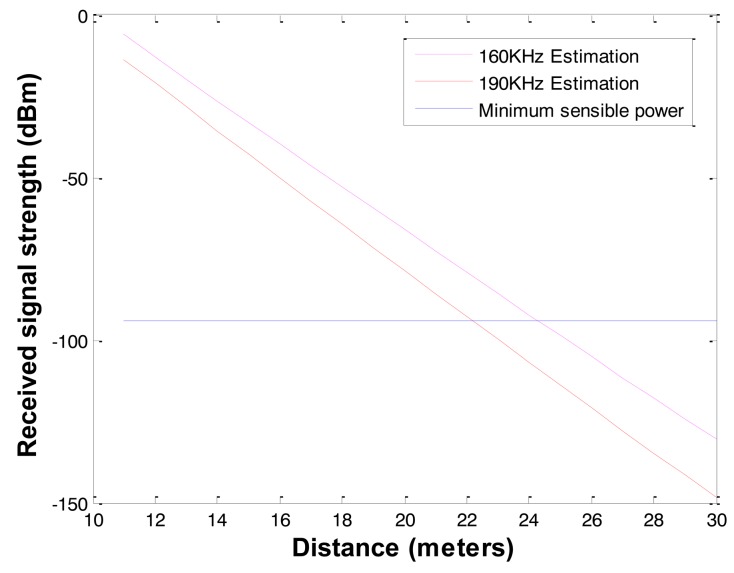
Underground radio signal attenuation with 160 kHz and 190 kHz.

To overcome high signal attenuation or increase communication radius in soil, three methods can be used: (1) high transmission power, (2) high gain antenna, and (3) low radio frequency. In case of high transmission power and high gain antenna, the benefit of extending communication radius is not enough to design practical underground applications. If the underground sensors use a low frequency, the communication radius can be extended more efficiently. There are candidates of low frequency bands for underground communication such as LowFER (Low-Frequency Experimental Radio) of 160 ∼ 190 kHz in the United States and Canada, and low frequency ISM (industrial, scientific and medical) bands of 6.78 MHz and 13.56 MHz. ISM bands are radio bands reserved internationally for the use of radio frequency for industrial, scientific and medical purposes. LowFER is a license-free form of two-way radio communications practiced on frequencies below 500 kHz [24]. The proposed propagation model is generic and applicable to a wide range of frequencies (1 MHz ∼ 2.5 GHz) besides the one used by the current wireless sensors operating on 2.4 GHz and 433 MHz [23]. Based on Equation (2), we can achieve 1.3 ∼ 2 m communication with 6.78 MHz and 13.56 MHz ISM bands, and 20 ∼ 25 m communication with 160 ∼ 190 kHz LowFER bands even in wet clay underground with 1 Watt Tx power and the same MICA's antenna gain as shown in Figures 3 and 4.

As an experimental platform, the USRP (Universal Software Radio Peripheral) systems are configured working on low frequency bands for underground communications [18]. USRP is an experimental platform of a GNU radio or software-defined radio (SDR) system where components that have been typically implemented in hardware (e.g., mixers, filters, amplifiers, modulators, demodulators, detectors, *etc.*) are instead implemented by means of software on a personal computer or embedded computing devices [25]. Using two USRP mother boards equipped with BasicTX/RX and LFTX/RX daughter boards, the communication systems working on low frequency bands are established in [18]. The BasicTX and BasicRX boards are designed for use with external RF frontends operating from 1 to 250 MHz frequency bands. LFTX and LFRX are similar to the BasicTX and BasicRX, but the operating frequency bands are from DC to 30 MHz with differential amplifiers and low pass filters. The USRP E100/E110 [26] which has an embedded processor with BasicTX/RX or LFTX/RX boards can be used for a low frequency wireless signal network platform.

## 4. Soil Properties Affecting Received Signal Strength

The received signal strength is affected by physical soil properties (e.g., soil density) and temporally dynamic variables (e.g., soil water content, salinity, soil temperature, *etc.*) which are affecting the electric conductivity and permittivity of the soil. To evaluate the variation of the received signal strength and apply the changing electric conductivity to the proposed theoretical model, the electric conductivity of the soil was measured for different water contents and salinity based on the American Society for Testing and Materials (ASTM) G187 standard [27] and the results are shown in Table 1. The experimental conditions for the measurements are described in Section 6, and the PVC box (shown in Figure 16) is used to evaluate the soil properties affecting the received signal strength. In the measurements, the first node (S1) was set as a sender and the other nodes (S2∼S6) are set as receivers recording the received signal strength.

### 4.1. Soil Gradation

**Figure 5.**
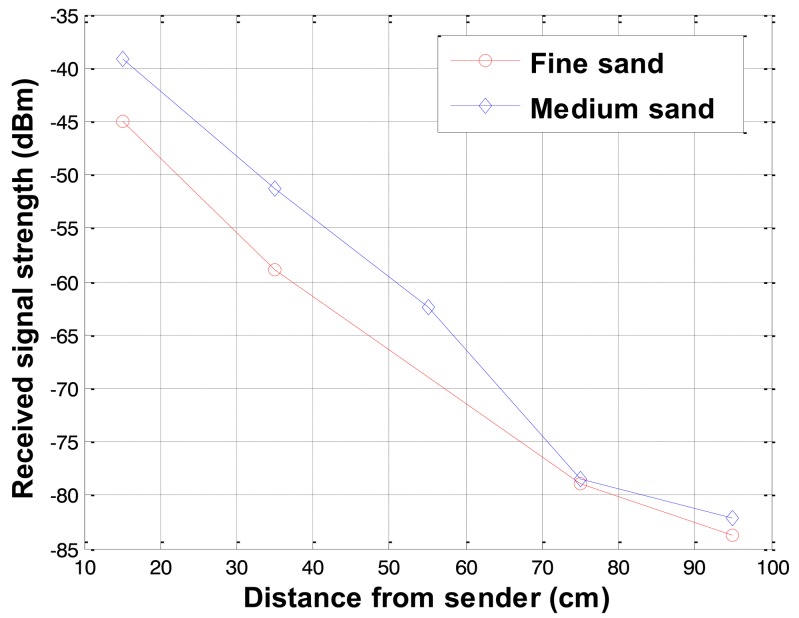
Received signal strength variations with soil gradation.

The received signal strengths in two types of sand with different gradations were compared. The comparisons of the received signal strength for the fine and medium sands at 12% water content are shown in Figure 5. The average particle size (*D*_50_) of fine and medium sand was 0.58 and 0.98 mm respectively. The received signal strength increases as the particle size in the soil increases. This may be attributed to the fact that, in the same soil type and condition, the soil with smaller particle size has higher electrical conductivity which imposes higher attenuation in the radio signal propagation.

### 4.2. Water Content

**Figure 6.**
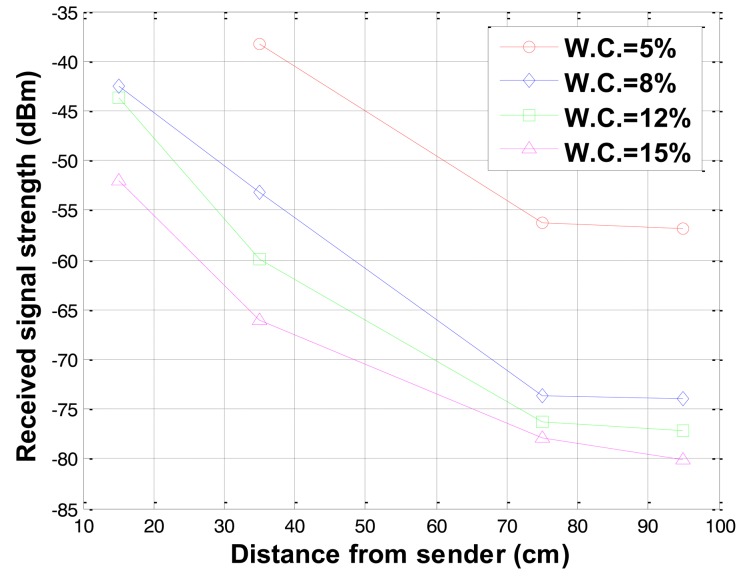
Received signal strength variations with water contents (W.C.).

To investigate the effects of soil water contents on the received signal strength, water contents were increased by 3–5% in each experiment. In the experiments using sandy type soils, the lowest water content level was 5% and the maximum water content level was 20%. In the case of 20% water content, the water was segregated from the soil grains. That is the maximum water content in the type soil sample used in the experiment. The increase in the water content increases the electric conductivity of the soil as presented in Table 1. The increased conductivity induces more attenuation on the underground radio propagation. The received signal strengths for different water contents were measured and the results for water contents of 5%, 8%, 12% and 15% are shown in Figure 6. As it can be seen in Figure 6, the received signal strength decreases as the water content increases.

### 4.3. Salinity

**Figure 7.**
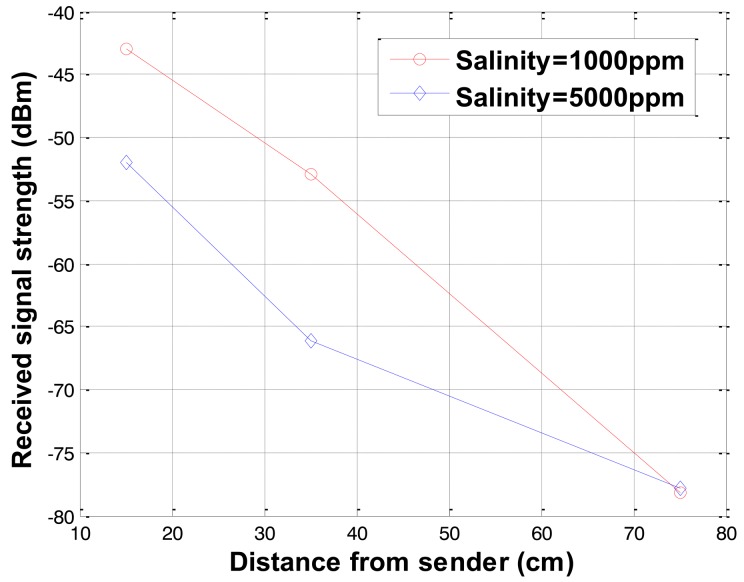
Received signal strength variations with salinity.

To demonstrate that the salinity can affect the received signal strength and wireless signal networks can distinguish different salinity levels, laboratory experiments were conducted. As the salinity of the soil increases, the electric conductivity of the soil increases (see Table 1). The received signal strengths for different salinity levels were measured and the results for salinity levels of 1,000 and 5,000 ppm at water content of 15% are shown in Figure 7 where the received signal strength decreases as the salinity increases.

### 4.4. Relative Density

**Figure 8.**
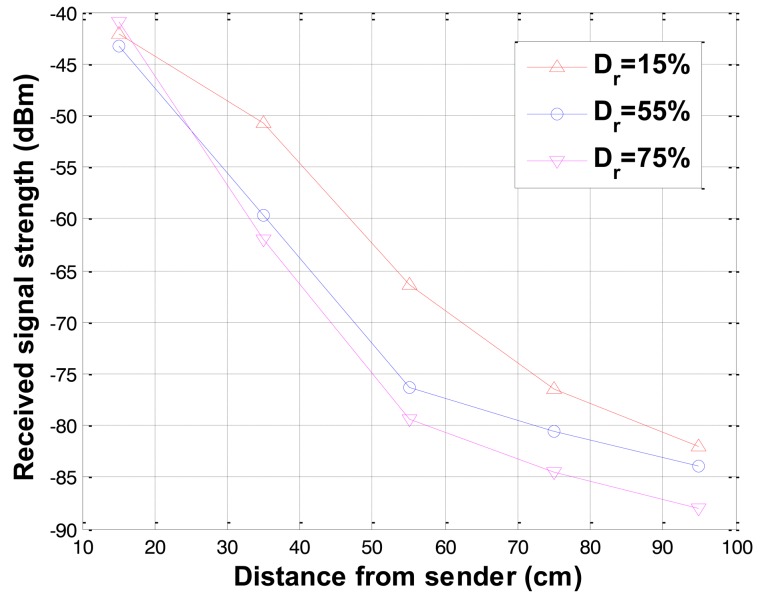
Received signal strength variations with relative density.

The received signal strengths for different relative densities (*D_r_*, an index that quantifies the state of compactness between the loosest and densest possible state of coarse-grained soils) were measured at constant water contents. The results for specimens of fine sand at relative densities of 15, 55, and 75% by compaction at constant water content of 12% are shown in Figure 8, where the received signal strength of the loose sand at 15% relative density is higher than the denser sands of 55% and 75% relative densities. The received signal strength decreases as relative density increases and the influence of signal attenuation is significant at longer distance.

### 4.5. Temperature

**Figure 9.**
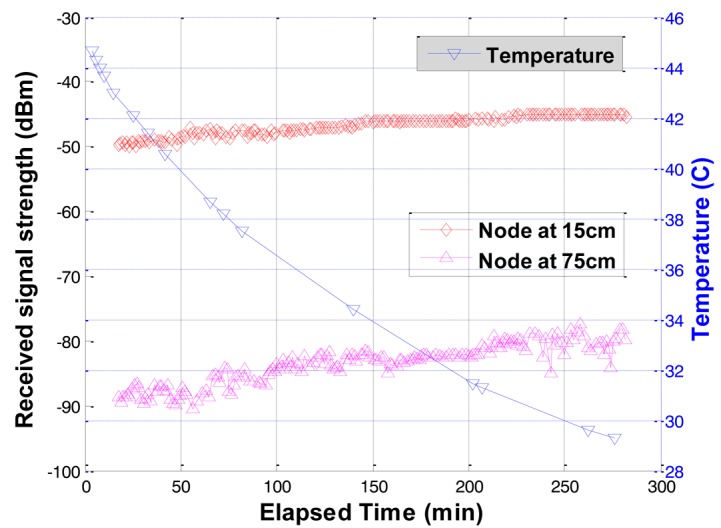
Received signal strength variations with temperature.

To investigate the effects of soil temperature on the underground radio propagation, the received signal strengths were measured in an experiment. The dry soil temperature decreased from 44.65 °C to 29.3 °C in the experiment. As shown in Figure 9, the received signal strength slightly increased as the temperature decreased. This trend can be explained by the slight decrease of electrical conductivity of soil when temperature decreases, and the negligible change of dielectric permittivity of soil for small to medium moisture contents (0∼32%) [28–30]. Thus, the increased received signal strength is attributed to the decrease of electrical conductivity as the soil temperature decrease.

In the wireless signal networks experiments, the received signal is affected by soil properties. In other words, the monitoring system based on the received signal strength can distinguish the changes on soil properties or detect subsurface events. From our measured data and based on existing works [18,31–33], the paper presents a detailed list on how radio propagation is affected by soil properties in subsurface communication environments. The summarized soil properties and the effects on the received signal strength are presented in Table 2. The Equation (2) can be expressed in logarithmic scale such as *P_r_* (*d*)*_dB_* = 10log*K* – 8.69*ad* – 20log*d*, where the changes on soil properties affect *a* and *K*. Based on the signal strength deviation, sensor nodes can detect the subsurface events as follows: *P_r_* (*d*,*t*)*_dB_* ≥ ζ × *P_r_* (*d*,*t* – Δ*t*)*_dB_* at the sensing time *t*, where ζ is the deviation criterion.

## 5. Subsurface Event Detection and Classification

The received signal strength can be used to detect and classify different subsurface events. The received signal strength information can be classified by minimum distance classifier using Bayesian decision theory. In the application of Bayesian theory to runtime wireless underground sensor networks, the computational power is an important factor to be considered because wireless sensor nodes have limited computational power. A MICAz sensor node has a low-power microcontroller (ATmega128L) which speed is 4 or 7 MHz [4]. The CPU-heavy computational works cannot be performed while it sends or receives data due to its hardware and software architecture. When sensor nodes send and receive data, the received signal strength information can be collected. The MICAz sensor node consumes power as follows: 0 dBm TX: 17.4 mA, −10 dBm TX: 11 mA, and receive mode 19.7 mA [4]. In all experiments, 0 dBm TX power is used. In this paper, a window-based minimum distance classifier is proposed considering the energy and computational efficiency for wireless sensor networks maintaining high accuracy with less computation. The window-based classifier for wireless signal networks has two steps: event detection (based on the deviation criterion) and event classification (minimum distance classification). With the event detection, the window-based classifier classifies geo-events on the event occurring regions that are called a classification window. The proposed window-based classification method in Section 5.2 is evaluated with a water leakage experiment in which the data has been measured in laboratory.

### 5.1. Event Detection (Window Selection)

Because the wireless sensor has limited battery and computational power, it is not reasonable to classify events at every sensing time. When the sensor node sends or receives data, it cannot compute the probabilities for the event classification. So, if the strength of the received signal does not change from previously collected data at a specific location, there is not new geo-hazard or event in the soil medium and the event classification is not required. Thus, it is important to detect the region of the event occurring with a simple classification such as two-category case (*ω*_1_: event, *ω*_2_: no-event) in which *ω*_1_ can be assigned to binary value 1 and *ω*_2_ to binary value 0. The subsurface event *ω*_1_ at *k-th* position (1 ≤ *k* ≤ *N*) can be detected by the signal strength deviation from existing *M* sample average at the *n-th* sensing time *t_n_* as follows:

(3) $$\left|x_{k}(t_{n})-\left[\sum_{j=n-1}^{n-M}x_{k}(t_{j})\right]/M\right|\geq \varsigma$$

where *ς* is the deviation criterion. The deviation criterion can be empirically decided (ex, 3 ∼ 5 dBm) based on the measured data which has small variation in soil.

### 5.2. Event Classification on Selected Window

There are *N* positions to sense geo-events in underground wireless signal networks. Let {*ω*_1_, *ω*_2_,…,*ω_c_*} be the finite set of *c* states of events. *P*(*ω_j_*) describes the prior probability that the event is in state *ω_j_*. The variability of a measurement in probabilistic terms is expressed as *x* which is considered a random variable whose distribution depends on the state of event which is expressed as *p*(*x*∣*ω_j_*). If we have an observation *x* for which *P*(*ω_j_*∣*x*) is greater than *P*(*ω_j_*∣*x*), there would be a higher possibility that the true state of the event is *ω_i_*. Thus, choosing *ω_i_* minimizes the probability of error. In the classification with more than one measurement, the scalar *x* is replaced by the feature vector *x⃗*, where *x⃗* is in an *N*-dimensional Euclidean space *R^N^*. The posterior probability *P*(*ω_i_*∣*x⃗*) can be computed from *p*(*x⃗*∣*ω_i_*) by Bayes formula:

(4) $$P(\omega_{i}|\vec{x})=\frac{p(\vec{x}|\omega_{i})P(\omega_{i})}{p(\vec{x})}$$

where $p(\vec{x})=\sum_{j=1}^{c}p(\vec{x}|\omega_{j})P(\omega_{j})$.

The Bayes formula shows that by observing the value of *x⃗* we can convert the prior probability *P*(*ω_j_*) to the *a posteriori* probability *P*(*ω_i_*∣*x*) - the probability of the state of event being *ω_i_* given that feature value *x⃗* has been measured. *p*(*x⃗*∣*ω_i_*) is called likelihood of *ω_i_* with respect to *x⃗*. The Bayes decision rule emphasizes the role of the posterior probabilities, and the evidence factor *p*(*x⃗*) is unimportant as far as making a decision is concerned. To represent event classifiers, a set of discriminant functions *g_i_*(*x⃗*) is used, where *i* = *i*,…,*c*. The classifier is said to assign a vector *x⃗* to class *ω_i_* if *g_i_*(*x⃗*) > *g_j_*(*x⃗*) for all *j* ≠ *i*. For the minimum error rate classification, discriminant functions are defined as follows [34]:

(5) $$\begin{array}{c} g_{i}(\vec{x})≜P(\omega_{i}|\vec{x})=\frac{p(\vec{x}|\omega_{i})P(\omega_{i})}{\sum_{j=1}^{c}p(\vec{x}|\omega_{j})P(\omega_{j})}\\ ≜p(\vec{x}|\omega_{i})P(\omega_{i})\\ ≜\ln p(\vec{x}|\omega_{i})+\ln P(\omega_{i}) \end{array}$$

where $\sum_{j=1}^{c}p(\vec{x}|\omega_{j})P(\omega_{j})$ does not affect on the decision and can be ignored.

The log scale expression of discriminate functions does not affect the decision as well, and will be used to develop a minimum distance classifier. The measured received signal of wireless sensors is assumed to be independent and normally distributed [35]. That is, each measurement is statistically independent and its probability density function is normally distributed. Thus, the discriminant function can be evaluated with the densities *p*(*x⃗*∣*ω_i_*) which are multivariate normal-that is, *p*(*x⃗*∣*ω_i_*) ∼ *N*(*μ⃗_i_*, Σ*_i_*) where *μ⃗_i_* is the mean vector and Σ*_i_* is the covariance matrix. The general multivariate normal density in *N* dimensional is written as:

(6) $$\text{s}\;p(\vec{x})=\frac{1}{{\left(2\pi \right)}^{N/2}{\left|\sum \right|}^{1/2}}\exp\;\left[-\frac{1}{2}{\left(\vec{x}-\vec{\mu }\right)}^{t}\sum^{-1}(\vec{x}-\vec{\mu })\right]$$

where *x⃗* is *N*-dimensional column vector, *μ⃗* is the N-dimensional mean vector, Σ is the *N-by-N* covariance matrix, and |Σ| and Σ^−1^ are its determinant and inverse, respectively [34]. With multivariate normal density, the discriminant function is express as follows:

(7) $$g_{i}(\vec{x})=-\frac{1}{2}{\left(\vec{x}-{\vec{\mu }}_{i}\right)}^{t}\sum^{-1}(\vec{x}-{\vec{\mu }}_{i})-\frac{M}{2}\ln2\pi -\frac{1}{2}\ln(\sum_{i})+\ln P(\omega_{i}).$$

when the features are statistical independent and each feature has the same variance *σ*^2^, the covariance matrix is expressed as Σ*_i_* = *σ*^2^I where I is the identity matrix. Then, the discriminant functions are simplified as follows [34]:

(8) $$g_{i}(\vec{x})=-\frac{{\left\|\vec{x}-{\vec{\mu }}_{i}\right\|}^{2}}{2\sigma^{2}}+\ln P(\omega_{i})$$

where ║•║ denotes the Euclidean norm, that is:

(9) $${\left\|\vec{x}-{\vec{\mu }}_{i}\right\|}^{2}={\left(\vec{x}-{\vec{\mu }}_{i}\right)}^{t}(\vec{x}-{\vec{\mu }}_{i}).$$

**Figure 10.**
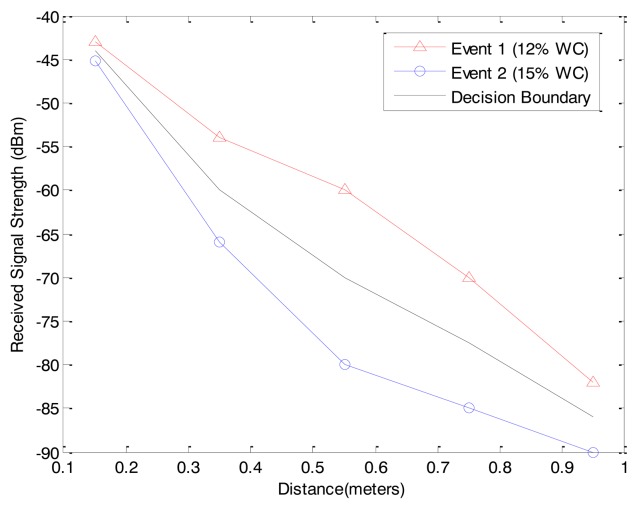
An example of decision boundary of minimum distance classifier for water leakage event.

If the prior probabilities *P*(*ω_i_*) in the log scale expression are the same for all *c* classes, the ln *P*(*ω_i_*) term becomes unimportant additive constant that can be ignored. In this case, the optimum decision rule can be a minimum distance classifier. To classify a feature vector *x⃗*, we measure the Euclidean distance ║*x⃗* − *μ⃗_i_*║ from each *x⃗* to each of the *c* mean vectors, and can assign *x⃗* to the category of the nearest mean. The subsurface events can be classified based on the training data (*μ⃗_i_*) and measured received signal strengths (*x⃗*). The training data can be generated from empirical data or theoretical estimation such that the event *ω_i_* has training data *μ⃗_i_*. Then, the measurement *x⃗* can be classified to the event *ω_i_* which has the minimum distance between *x⃗* and *μ⃗_i_* or the highest probability that the true state of the event is *ω_i_*. Based on the minimum distance classifier, the decision boundary can be calculated. For example, the received signal strengths of two events (two different water content measurements using MICAz where 12% is measured water content in normal condition and 15% is the water content after the water leakage event in Section 6.2) at 5 positions and the connected decision boundary of minimum distance classification for 5 positions are shown in Figure 10.

After detection of a subsurface event shown in Section 5.1, the event is classified based on the selected window. The minimum-error-rate classification can be achieved by using the discriminant functions with *x⃗* = {*x_k_*} ∈ ψ where ψ is a set of *W* features in classification window which size is *W*. The window based selection reduces the error rate of the classification. Suppose that we observe a particular *x_k_* and we contemplate taking decision *α_i_*. If the true state of the event is *ω_j_*, the classification has the loss defined as λ(*α_i_*∣*ω_j_*). Because *P*(*ω_j_*∣*x_k_*) is the probability that the true state of the event is *ω_j_*, the expected loss associated with taking decision *α_i_* on selected window, *R_w_*, is:

(10) RW(αi∣x→)=∑k=1W∑j=icλ(α∣iωj)P(ωj∣xk)≤∑k=1N∑j=icλ(α∣iωj)P(ωj∣xk)=RN(αi∣x→N)

where *R_N_* is the expected loss of whole range and *W* ≤ *N*. The expected loss of window classifier *R_W_* is lower than the expected loss of whole range classifier s *R_N_* as shown in Equation (10). For example, the minimum distance classifier on all sensing positions has a higher error rate due to the variation of received signal strength in no event region.

**Figure 11.**
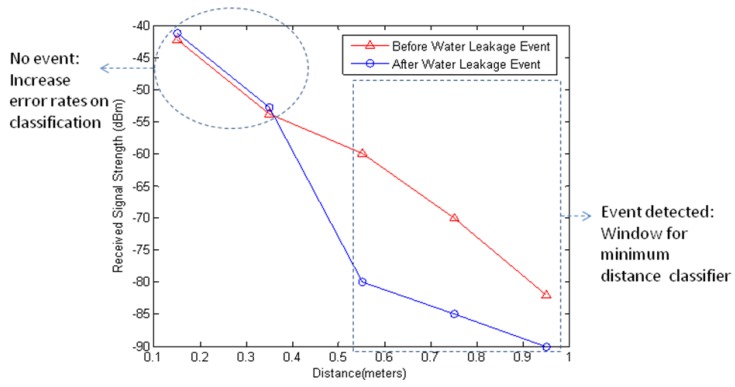
A window for minimum distance classifier.

Figure 11 shows the window for minimum distance classifier and error prone points from the water leakage event in Section 6.2.2. The window-based computation has lower computational cost than whole range computation. In other words, the computational cost of whole range minimum distance classifier is *O*(*cN*) where *c* is the number of classes and *N* is the number of features, whereas the cost of the window based minimum distance classification is *O*(*cW*) where *W* is the window size and *W* ≤ *N*.

## 6. Performance Evaluations

The concept of wireless signal networks for subsurface event detection and classification is validated with widely used sensor nodes (*i.e.*, MICAz) because of their stability for the measurements of received signal strength. The MICAz sensor nodes are with the CC2420 RF transceiver which includes a digital direct sequence spread spectrum baseband modem. The operating frequency was configured to be 2.48 GHz which is Zigbee channel 26 and non-overlapping with 802.11 b (WiFi). All wireless sensor nodes are calibrated and selected to be working in 1 ∼ 2 dBm error bounds on the received signal strength measurement. A wireless sensor node (sender) sends a packet periodically (at every 15 seconds for subsurface event detection experiment), and the receivers sample the received signal strength from the received packets and store the date on the flash memory in which data can be retrieved in the laboratory with serial or ethernet programming boards connected to the desktop.

### 6.1. Subsurface Event Detection

**Figure 12.**
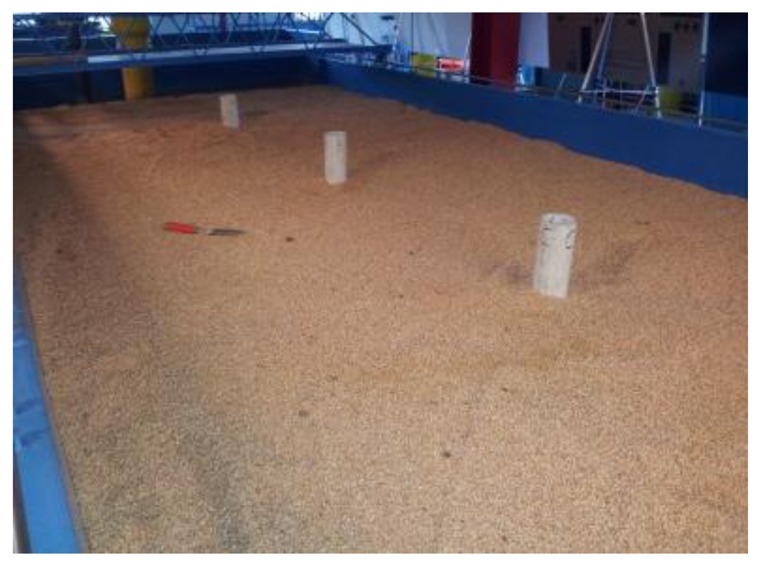
Large soil box for experiments of subsurface event detection.

The experiments were designed to demonstrate the functionality of the WSiN to detect transient changes in host soil within the network domain. Using the decision rule introduced in Section 5.1, the subsurface events can be detected based on the empirically or theoretically determined deviation criterion *ζ* (ex, 3–5 dBm). Three subsurface event experiments were conducted to generate transient changes within host soil mass (coarse sand, *D*_50_ = 3.3 mm where *D_x_* is the diameter of the soil particles for which *x* of the particles are finer) in the large soil box shown in Figure 12. These events were (1) water intrusion, (2) relative density change, and (3) relative motion.

#### 6.1.1. Detection of Water Intrusion

**Figure 13.**
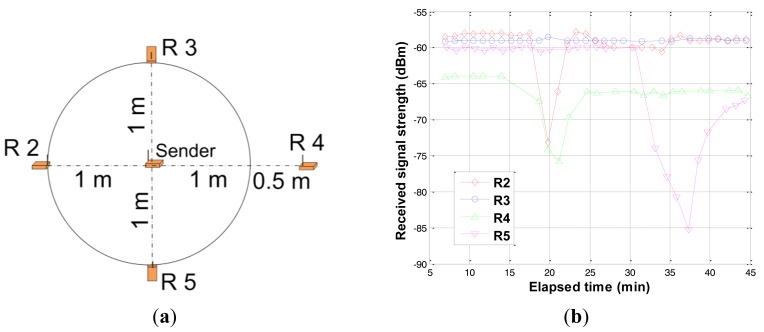
Experiment of water intrusion detection. (**a**) Configuration for water intrusion; (**b**) Results of water intrusion detection.

Water intrusion events were simulated by gradually injecting water between the nodes. Figure 13(a, b) show the network system configuration and results of water intrusion experiment respectively. All transceivers were buried at a depth of 20 cm at the locations shown in Figure 13(a). The initial water content of the soil was 6%. The first event was started at 17 minutes (elapsed time) where water was injected between the sender and node R2 (Receiver 2). The next event was started at 19 minutes where water was injected between the sender and node R4. The last event was started at 32 minutes where water was injected between the sender and node R5. As shown in Figure 13(b), there is a significant decrease in the received signal strength right after the water injection in all three cases. However, there is no change in the received signal strength at node R3 (fairly constant) which was not located in the region of events. Average water content of soil located between the sender and R2/R4/ R5 after the injection event was determined as 12%, 11%, and 16% respectively. The increased water content of soil increased the permittivity and electrical conductivity of the medium, resulting in decrease of received signal strength. It is important to note that the magnitude of signal depletion is proportional to the magnitude of change in the water content (comparing nodes R2 and R5). Also, since node R4 was located at farther distance than the other nodes, it has a lower value of received signal strength.

#### 6.1.2. Detection of Relative Density Change

**Figure 14.**
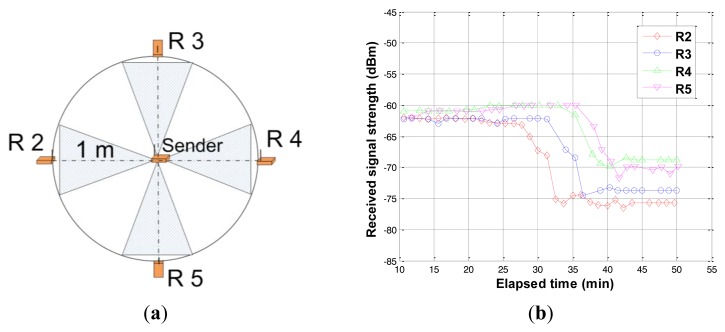
Experiment of relative density change detection. (**a**) Configuration for relative density change; (**b**) Results of relative density change detection.

Relative density change experiment through soil compaction was conducted by applying vibration at the soil surface using magnetic vibration equipment. All transceivers were buried at a depth of 20 cm with network configuration as shown in Figure 14(a). Each shaded area between the sender and receivers (starting from node R2 toward R5) in Figure 14(a) were compacted for about 2 minutes starting at 27, 32, 36, and 38 minutes. The water content of the soil was 5%. As shown in Figure 14(b), the change in the compactness of soil is detected by marked drops in signal strengths at all nodes. As shown in the parametric test results in Table 2, the increase in soil density reduces the signal strength. The drop in received signal strength may be attributed to the increase in electrical conductivity of the medium. Compacted soil has more surface particle contacts compared to the loose soil and provides more electron flow resulting in higher electrical conductivity of the compacted media. Consequently, the received signal strength decreases as the soil compactness (relative density) increases.

#### 6.1.3. Detection of Relative Motion

**Figure 15.**
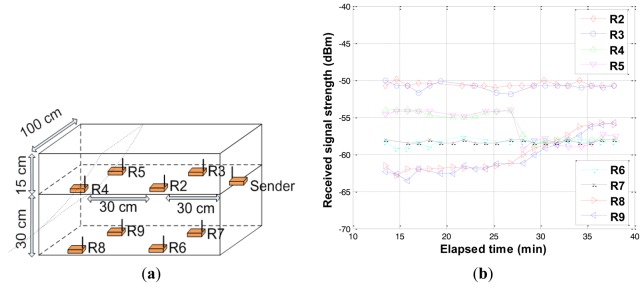
Experiment of relative motion detection. (**a**) Configuration for relative motion; (**b**) Results of relative motion detection.

In this experiment, the sender and the nodes R2, R3, R4, and R5 were located in the same plane at a depth of 15 cm from the surface, and the nodes R6, R7, R8, and R9 were located at the same plane at a depth of 45 cm from the surface as shown in Figure 15(a). The soil mass was held using a wooden plate in the front while the other sides remained continuous. The event started at 27 minutes by removing the wooden plate. Relative motion experiment results are shown in Figure 15(b). The received signal strength at nodes R2, R3, R6, and R7 remained fairly constant. Since neither the physical properties of the soil nor the distance between the sender and receiver changed within these regions, no change was recorded in the received signal strength. At nodes R4 and R5 that were directly affected by the event, the received signal strength dropped and remained constant as shown in Figure 15(b). The drop is attributed to a combination of different factors including change in the distance between the sender and receivers, antenna orientation, and density of soil between the sender and receivers. In contrast, an increase in the received signal strength at nodes R8 and R9 was detected. This increase may be attributed to the removed mass of soil initially located on top of these nodes. The important point to notice is that the event could be detected through changes in the received signal strength of the nodes within the network domain.

### 6.2. Subsurface Event Classification

**Figure 16.**
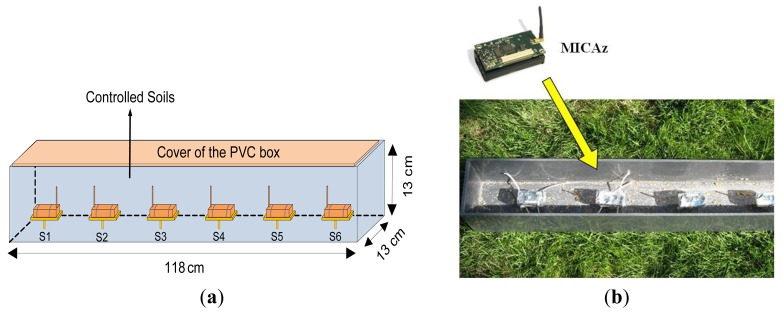
Details of designed PVC box and sensor installation. (**a**) Designed PVC box to install sensors (one transmitter and receivers); (**b**) PVC box with sensors before soil filling.

For the sake of better and more accurate control on the soil properties comparing with the large soil box shown in Figure 12, a small plastic (PVC) box with dimensions 118 × 13 × 13 cm was made as shown in Figure 16(a) and 16(b) and filled with controlled soils. In the experiment, physical soil properties (e.g., soil density) and temporally dynamic variables (e.g., soil water content, salinity, soil temperature, *etc.*), which affect the electric conductivity and permittivity of the medium, were controlled. To evaluate the proposed classifier, we applied the classifier into a water leakage experiment where soil properties were controlled where the soil is fine sand (*D*_50_ = 0.58 mm).

In the experiment, sensors (S2–S6) sent a packet at every 60 seconds and the receiver node (S1) calculated the received signal strength based on the received packet. Based on the collected received signal strength information, the node S1, which can be a sink node or an intermediate node that can send or receive data, estimates the location of water leakage event and classifies the water contents on the soil media between the transmitters and receiver. In the water leakage experiment, the soil in the box contained 12% water content before the water leakage event. The water leakage event was start at 26 minutes after experiment start by injecting water into the regions between 40cm and 60cm from the first node (S1). After the water leakage event, the average volumetric water content of the soil in the box was 15%.

#### 6.2.1. Reference Data Generation

**Figure 17.**
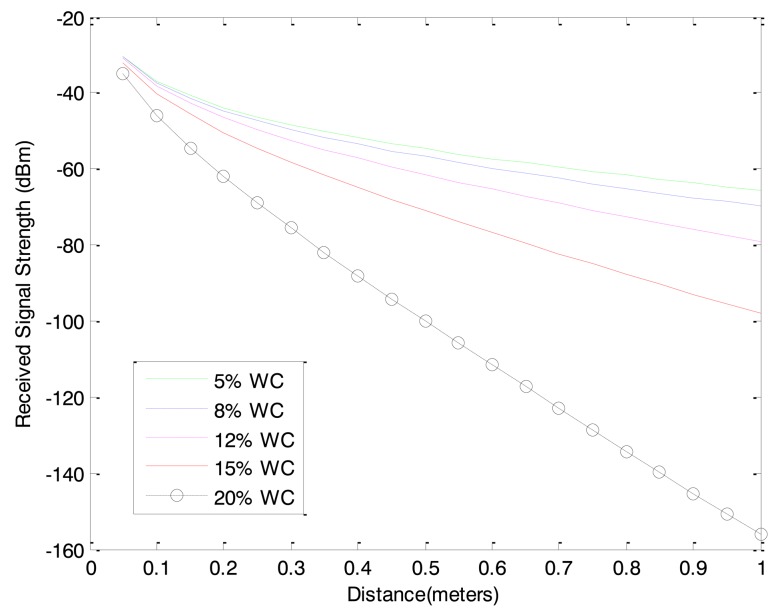
Received signal strength estimation with different water content.

In the evaluation, we classify the geo-events based on training data which can be the measured data or theoretically estimated data. Using the accurate underground radio propagation model introduced in [23], we generate the estimated received signal strength with different water content as the reference data (training data for the event classification) which are shown in Figure 17. In the estimation using Equation (2), the electric conductivity values of the soil in all experiments were measured as shown in Table 1 and the relative permittivity of the soil is estimated between 19–30 based on [36].

#### 6.2.2. Event Classification of Water Leakage Experiment

**Figure 18.**
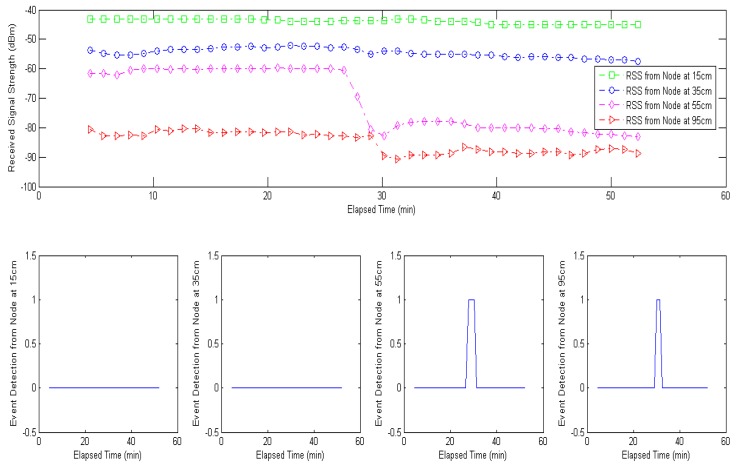
RSS measurements in water leakage experiment and event detection signals from different location (nodes at 55 cm and 95 cm) generate event detection signal.

Figure 18 shows the time evolution of the received signal strength when the water leakage event was conducted. With the water leakage event at 26 minutes, the nodes at 55 cm and 95 cm have low received signal strength due to high signal attenuation from increased water content. As a result, the node at 55 cm generates event detection signal at 26, 27, and 28 minutes and the node at 95 cm generates event detection signal at 28 and 29 minutes based on the decision rule, where the deviation criterion *ζ* is 3 (dBm) and the previous sensing time *M* is 3 (minutes). Thus, the minimum distance classification will be conducted only from 26 minute to 29 minute.

**Figure 19.**
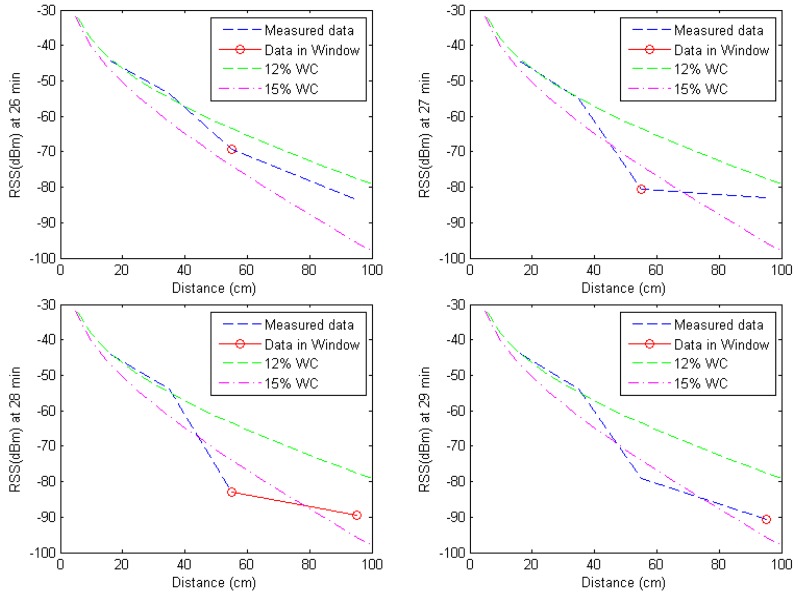
Event classification based on received signal strength between 26 and 29 minutes (detected events at 26, 27, and 28 minutes are classified as 15% water leakage event).

When events are detected, the window-based minimum distance classifier classifies the event based on the measured data of the detected region by calculating the minimum distance between the detected event and the reference data using Equation (8). Figure 19 shows the received signal strength between 26 and 29 minutes where the event detection signals are generated. In Figure 19, the red circle represents the data in the classification window that can be used in the minimum distance classification. The detected events at 26, 27, 28, and 29 minutes are classified as 15% water leakage event based on the window-based minimum distance classifier as shown in Figure 19. In case of whole-range minimum distance classification, the detected events are classified as 12% water leakage event at 26 and 27 minutes and 15% water leakage event at 28 and 29 minutes. We compared window-based minimum distance classification and whole-range minimum distance classification and summarized the results in Table 3. The proposed method generates four event detections during the water leakage experiment. When the events are detected, the window-based minimum distance classification detects the leakage event correctly with 100% accuracy; where as the whole range minimum distance classification has 50% accuracy.

The window-based minimum distance classification has 68% less computation than the whole range minimum distance classification in the experiment.

## 7. Conclusions

The received signal strength information of underground sensors is used to characterize the geo-event in the proposed underground wireless signal network. This paper presents a detailed list of soil properties affecting underground radio propagation with experimental data. To evaluate the concept of WSiNs for subsurface event detection, three event detection experiments (water intrusion, relative density change, and relative motion) were conducted using MICAz. In the paper, the window-based minimum distance classifier is proposed to detect and classify geo-events of wireless underground sensor networks. The window-based minimum distance classifier accurately detects geo-events and classifies the water leakage event into different water contents. From the theoretical analysis and experiment, the proposed window-based minimum distance classifier has less computation and higher accuracy than whole range minimum distance classifier. In the future, we will conduct experiments for geo-event detection and classification of heterogeneous conditions, such as simultaneous water leakage and land slide occurrence, and apply the proposed window-based minimum distance classifier to the heterogeneous geo-events.

## Figures and Tables

**Table 1.** Electric conductivity of the soil used in received signal strength measurements.

**Soil Type**	**Water Content (%)**	**Salinity (ppm)**	**Electric Conductivity (mS/m)**
Fine Sand	5	1,000	23.42
		5,000	26.67
	8	1,000	33.16
		5,000	37.61
	12	1,000	65.36
		5,000	86.96
	15	1,000	123.61
		5,000	146.05
	20	1,000	238.66
		5,000	283.77

**Table 2.** Soil properties affecting the received signal strength in underground applications.

**Soil Properties**	**Effects on received signal**
Soil Gradation	Particle size ↑ → received signal ↑
Water content	Water content ↑ → received signal ↓
Salinity	Salinity ↑ → received signal ↓
Relative density	Relative density ↑ → received signal ↓
Temperature	Temperature ↓ → received signal ↑

**Table 3.** Comparisons of Minimum Distance Classifier (MDC).

**Time (min.)**	**Event Detection**	**Window-based MDC**		**Whole Range MDC**	
	
		**Generated Event**	**Classified Event**	**Generated Event**	**Classified Event**

26	Node at 55 cm	15% WC	15% WC	15% WC	12% WC
27	Node at 55 cm	15% WC	15% WC	15% WC	12% WC
28	Nodes at 55, 95 cm	15% WC	15% WC	15% WC	15% WC
29	Node at 95 cm	15% WC	15% WC	15% WC	15% WC
